# EGG: Accuracy Estimation of Individual Multimeric Protein Models Using Deep Energy-Based Models and Graph Neural Networks

**DOI:** 10.3390/ijms25116250

**Published:** 2024-06-06

**Authors:** Andrew Jordan Siciliano, Chenguang Zhao, Tong Liu, Zheng Wang

**Affiliations:** 1 Department of Computer Science, University of Miami, 1365 Memorial Drive, Coral Gables, FL 33124, USA; ajs550@miami.edu (A.J.S.); tong.liu@miami.edu (T.L.); 2 Computer Information Sciences Department, St. Ambrose University, 518 W. Locust Street, Davenport, IA 52803, USA; zhaochenguang@sau.edu

**Keywords:** structural bioinformatics, bioinformatics, neural networks

## Abstract

Reliable and accurate methods of estimating the accuracy of predicted protein models are vital to understanding their respective utility. Discerning how the quaternary structure conforms can significantly improve our collective understanding of cell biology, systems biology, disease formation, and disease treatment. Accurately determining the quality of multimeric protein models is still computationally challenging, as the space of possible conformations is significantly larger when proteins form in complex with one another. Here, we present EGG (energy and graph-based architectures) to assess the accuracy of predicted multimeric protein models. We implemented message-passing and transformer layers to infer the overall fold and interface accuracy scores of predicted multimeric protein models. When evaluated with CASP15 targets, our methods achieved promising results against single model predictors: fourth and third place for determining the highest-quality model when estimating overall fold accuracy and overall interface accuracy, respectively, and first place for determining the top three highest quality models when estimating both overall fold accuracy and overall interface accuracy.

## 1. Introduction

The process by which a sequence of amino acids (primary structure) converges to form a three-dimensional (tertiary) structure, known as protein folding, has been of great interest across scientific communities. The three-dimensional structure of proteins is crucial to understanding how they function, interact with, and impact biological systems [1]. Experimental methods (e.g., X-ray Crystallography and Nuclear Magnetic Resonance) of obtaining the native structure of a protein molecule are time-consuming and costly [1,2]. Predicting the tertiary structure from a given primary structure has the potential to positively influence biological and medical research significantly [1,3]. Multiple chains can merge to form a protein complex over inter-subunit interfaces, known as the quaternary structure. Predicting the quaternary structure is becoming increasingly important, as vital cellular processes heavily involve protein complexes [4].

The accuracy estimation of predicted protein models (EMA) is a crucial step for determining their respective utilities. Many types of quality scores, with diverse purposes, can be generated for a given model. Accuracy is defined as the deviance of the predicted model from its native structure. EMA can produce a variety of these quality scores, which help us to determine the strengths and weaknesses of each model, indicating their most effective use case or cases. The Critical Assessment of Protein Structure Prediction 15 (CASP15) experiment exclusively performed EMA upon multimeric protein models.

Deep learning has been used to perform accuracy estimation of both single and multiple-chain (multimers) proteins. Processing not only the individual properties of each residue, but also the interactions between them, is vital to a successful EMA algorithm. Graph neural networks (GNNs) are a pioneering subset of deep learning that takes a graph as input and directly account for the relations between nodes (edges). GNNs allow for arbitrary-sized input graphs, and traditionally output a topologically equivalent graph to the input. Cutting-edge architectures like graph transformers [5,6] process the relationships between nodes (edges) using attention mechanisms [7]. Incorporating attention into message-passing frameworks improves the GNN’s ability to determine the importance of nodes and or edges, given their context, through learned attention coefficients. GNNs in recent years have excelled in regression problems, and have been used in EMA for both single and multiple-chain proteins [8,9,10,11].

The success of a deep learning algorithm depends largely upon the training data. Nodes and edges must contain meaningful and indicative feature vectors for GNNs to perform their best. Energy scores, an indicator of molecular stability, are commonly used as input features to deep learning EMA models. It is widely accepted that the native conformations of proteins are associated with points of minimum free energy [12]. Energy functions, which produce energy scores, aim to resemble the free energy quantitatively (e.g., the Rosetta [13] energy function). Energy scores have been used to rank protein models (or complexes), assuming the most accurate models are the most molecularly stable.

Deep neural networks can be trained to emulate an energy function, denoted as energy-based models (EBM). EBMs produce associated energy scores, where smaller values indicate a higher likelihood of the input being correct and larger values indicate a lower likelihood of the input being correct. Correctness is arbitrary, and can have different meanings given different contexts. For example, the correctness of a protein could indicate a high molecular stability, whereas the correctness of an image could indicate the likelihood the image matches a certain label or falls within a specific distribution of images.

Energy-based graph convolutional networks (EGCN) [14] have been deployed for predicting the qualities of protein docking models, a similar task to that of EMA for protein-complex models. EGCN [14] was trained using the absolute difference between the predicted and target (binding energy) values as the loss function. Typically, energy-based models are trained without using the absolute difference or mean squared error loss functions, and instead utilize objective functions that minimize and maximize the output energy given true and adversarial inputs, respectively. Protein-EBM [15] used this approach to deploy and learn state-of-the-art energy-based models directly from protein structures (monomers), using the log-likelihood objective function.

Despite this, to the best of our knowledge, energy-based models have not been used to directly infer the qualities of multimeric protein models. In this paper, we present two approaches that use cutting-edge graph neural networks and novel energy-based models. Graphs are constructed based upon the geometry and topology of the input protein complex structure, where each node represents a residue and edges represent the spatial interactions between them. The EBMs were used to predict the quality of protein complex structures from the perspective of free-energy minimization. Our evaluation results demonstrate significant performance for discerning high- from low-quality computationally predicted multimeric protein models.

## 2. Results and Discussion

To compare our EBM and GNN models, we computed their respective mean absolute error (L1) and mean squared error (MSE) losses between the predicted and true accuracy scores of the given multimeric protein models, for both overall fold (TM-score) and interface (QS-score) accuracy scores. Both methods achieved comparable results, with only slight differences (see Table 1).

CASP15 ranking loss [16] is defined as the difference between the true top-ranked model and the top-ranked model determined by a predictor’s method. The group name APOLLO was the original model submitted to CASP15. The group names associated with our presented methods are denoted as EBM–{layer} and Regression–{layer}, with the layer being either “Transformer” or “MetaLayer”. Our models were trained as single-model predictors [8,17,18,19] and, therefore, we filtered all consensus-model predictors.

We found that our methods, on rare occasions, hit a plateau in the energy function, meaning the same prediction is made for all models of a given target. This is not the case for APOLLO, for which this issue was present in many of the CASP15 targets. We slightly modified the CASP15 ranking loss criteria in our blind test of CASP15 targets to address this issue. We only accept a top-ranked model from a given predictor if the predicted quality score is unique for all models in the given target. When these cases occur, we ignore this predictor’s prediction and treat it as if the ranking loss was not less than 0.1. This stricter criteria agrees well with the intention of these ranking metrics, as when multiple protein models are ranked at the highest quality, we cannot determine which to use in the real world. CASP15 evaluators considered all predictions, and treated the first model as the highest predicted quality in these cases, allowing for arbitrary rankings to be introduced.

**Figure 1:**
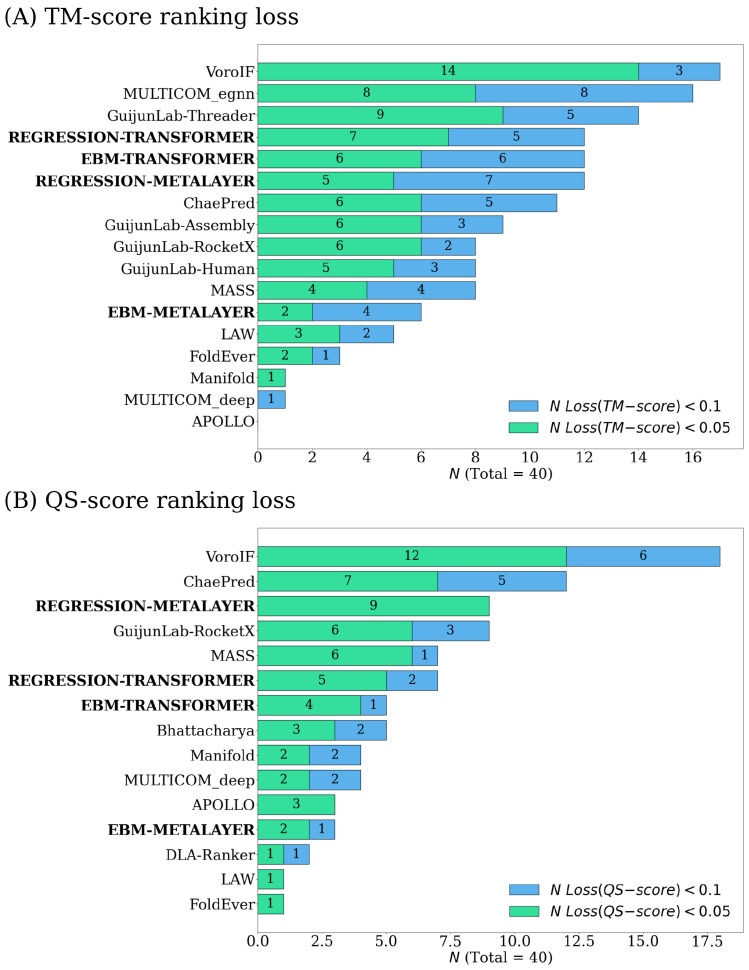
The ranking of CASP15 single-model methods based on loss. All 23 two-chain models (dimers) and 17 more-than-two-chain models released by CASP15 were evaluated for a combined total of 40 multimeric protein targets (x-axis) spanning 10,472 predicted models [16]. Loss refers to the CASP15 ranking loss [16] with included modifications to account for non-unique top predicted quality scores.

Our methods, when evaluated against these modified CASP15 ranking loss metrics, generally improved from their predecessor APOLLO (see Figure 1). The performance of our EBMs are comparable to the performance of the transformer backbones, but performed worse compared to the MetaLayer backbones. This suggests EBMs can complement the estimates produced by GNNs, but do not universally match their performance for accuracy estimation tasks. Our EBM–Transformer and Regression–Transformer ranked fifth and fourth, respectively, in the “TM-score ranking loss category”. Our Regression–MetaLayer ranked third in the “QS-score ranking loss category”. For individual target performance metrics with respect to CASP ranking loss, refer to the Supplementary Document (S2).

**Figure 2:**
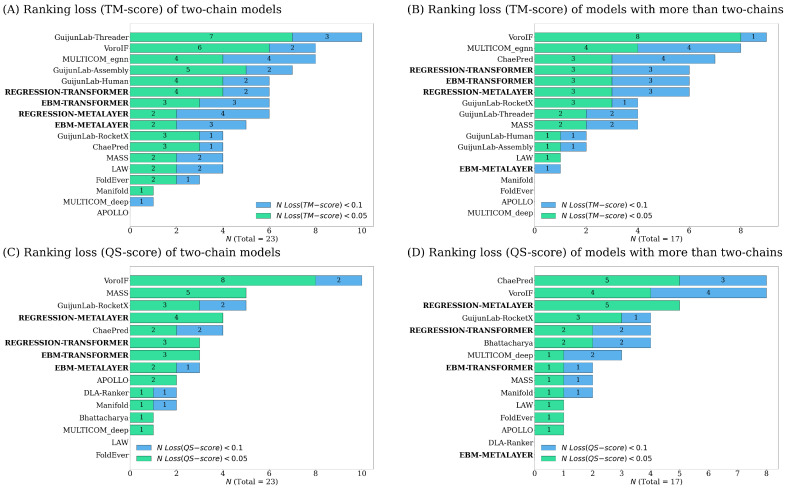
Ranking of all CASP15 single-model methods with different numbers of chains based on loss.

When analyzing the predicted models, it is vital to holistically view the accuracy estimates across multiple metrics. This reduces the probability of outliers obfuscating the potential utility of a model. To further investigate where our methods performed best, we ranked single model methods again using the CASP ranking loss, but against strictly two-chain and more-than-two-chain targets (see Figure 2). We see our EBM–MetaLayer improve four and seven places when evaluating two-chain compared to more-than-two-chain models for overall fold and interface scores, respectively. For interface scores, our Regression-MetaLayer ranks fourth for two-chain and third for more than two-chain models, suggesting comparable performance independent of the number of chains. When estimating the overall fold accuracy, our Regression and EBM–Transformer architectures moved down in rankings for two-chain targets, suggesting stronger performance when evaluating more-than-two-chain proteins.

In addition to these aforementioned metrics, we implemented normalized discounted cumulative gain on the top three (NDCG@3) [20] ranked models per target.

(1) $$r_{i}=\left\{\begin{array}{cc} 1-{\left(Score\left(M\right)-Score\left(m_{i}\right)\right)}^{2} & \mathrm{if}\;Score(m_{i})\;\mathrm{is}\;\mathrm{unique}\\ 0 & else \end{array}\right.$$

(2) $$\mathrm{DCG}@3=\sum_{i=1}^{i=3}\frac{r_{i}}{log_{2}\left(i+1\right)}$$

For each position *i* in a given ranking, the relevance $r_{i}$, defined by Equation (1), is set as one minus the squared difference between the true quality score of the top-ranked model *M* and the model $m_{i}$. If a model at position *i* does not have a unique quality value, we set $r_{i}$ equal to zero. The formula used to compute the DCG@3 for a given target and predictor is defined by Equation (2). We divide the DCG@3 of the predictors by the DCG@3 of the true ranking of the models for normalization. In this way, the maximum achievable NDCG@3 for each target is one, and therefore the maximum total score any method could achieve is forty (one for each target).

**Figure 3:**
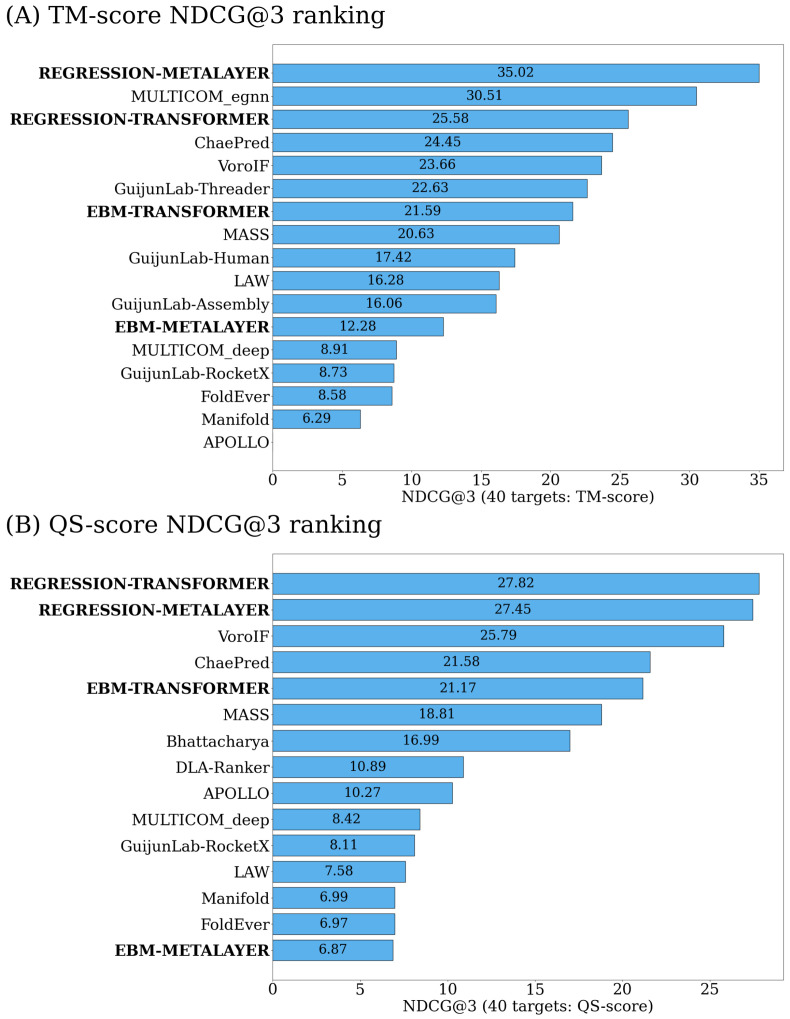
Ranking of all CASP15 single-model methods based on NDCG@3.

Figure 3 ranks the single-based methods upon the sum of their respective NDCG@3 values over all CASP15 targets. Our Regression–MetaLayer ranked first for overall fold accuracy, and our Regression–Transformer ranked first for overall interface accuracy. Furthermore, the majority of our methods (excluding our EBM–MetaLayer) achieved total scores greater than half of the greatest possible NDCG@3 score, across both the overall fold and the interface accuracy rankings. This indicates that the top three ranked models by the majority of our methods often contain suitable choices for higher quality models. For individual target performance metrics with respect to NDCG@3, refer to the Supplementary Document (S2).

**Figure 4:**
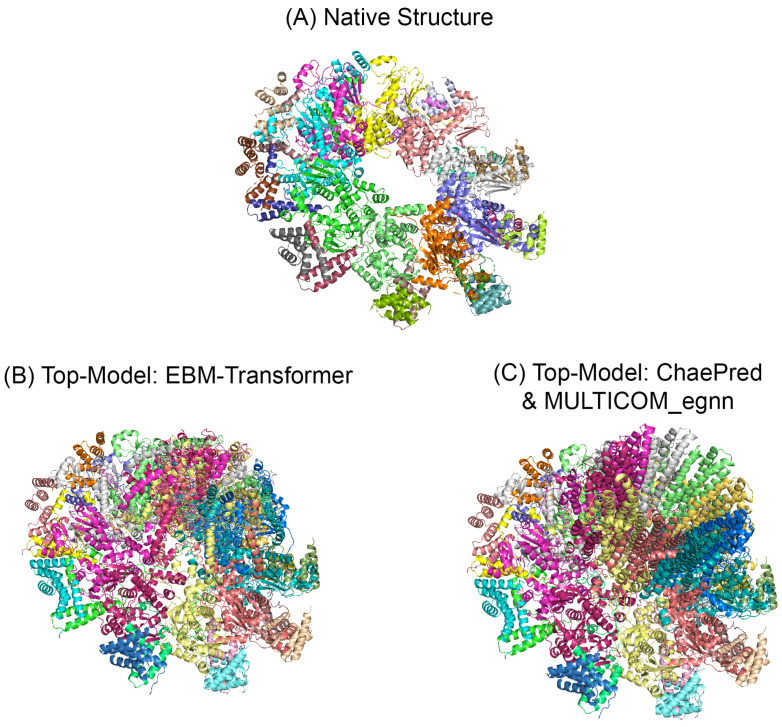
CASP15 Target H111 (overall fold accuracy) native structure (**A**) and top-ranked models by our “EBM–Transformer” (**B**) and “Chae Pred” and “MULTICOM_egnn” (**C**).

**Figure 5:**
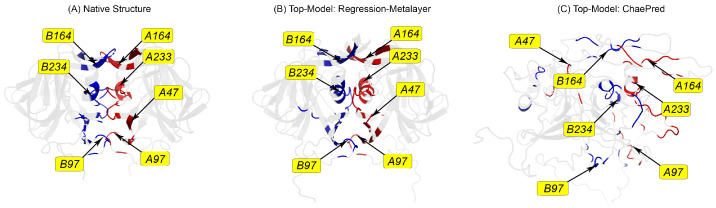
CASP15 Target T1123o (overall interface accuracy) with highlighted native interface residues on the native structure (**A**), the top-ranked model by “Chae Pred” (**C**) and by our “Regression–MetaLayer” (**B**).

We also see our respective methods order predicted models with a higher degree of precision than other top-ranked single model predictors on specific CASP15 targets (see Figure 4 and Figure 5). In Figure 4, the top-ranked model by “Chae Pred” and “MULTICOM_egnn” (C) had a TM-score of ≈0.696. The top-ranked model by our “EBM–Transformer” (B) had a TM-score of ≈0.982. Both models (Figure 4B,C) are aligned and superimposed with the native structure (Figure 4A) using PyMol [21]. In Figure 5, the interface residues are defined as residues within 8Å of any other residue from another chain. Residue positions are set as CB coordinates for all residues, except for GLY, which is set as CA. Both models (Figure 5B,C) are aligned and superimposed with the native structure (Figure 5A) using PyMol [21]. The top-ranked model by “Chae Pred” (Figure 5C) had QS-score ≈0.019. The top-ranked model by our “Regression–MetaLayer” (Figure 5B) had QS-score ≈0.549. It can be seen in Figure 5 that the arrangements of the interfacing residues selected by our methods highly match those of the native structure compared to the top model selected by “Chae Pred”.

## 3. Materials and Methods

### 3.1. Datasets

Our data comprised of homomer and heteromer models from CASP13-14 experiments, and approximately 10,000 protein complex structures from the protein databank [22], chosen before May of 2022, the start of CASP15. In this way, our methods had access to the same information as groups from CASP15, allowing for fair comparisons between them. Using PyRosetta [23], we further generated examples by performing perturbation upon these structures. Our data were randomly split by associated native pdb_id into training and validation sets of 23,313 and 2369 examples, respectively. The TM-score [24] and QS-best [25] were used as the target values for the overall fold score and overall interface score, respectively.

Each node in an input graph represents a residue in a multimeric protein model. ESM embeddings [26], including their mean, median, standard deviation, and variance, were used as parts of the node features. We also generated 83 additional node features, stemming from a total of six types: one hot encoding of the amino acid, position-specific scoring matrix created using PSI-BLAST [27] from a multiple sequence alignment, normalized Rosetta [13] energy scores, SOV_refine [28] scores for sequence-based and model-based secondary structures and solvent accessibility, fixed sequence-based sinusoidal positional encodings [7], and MASS [29] protein statistical potentials, including pseudo-bond angle potential, accessible surface potential at the atomic level, sequence separation-dependent potential, contact-dependent potential, relative solvent accessibility potential, and volume-dependent potential.

Edges were created for any residue pairs that have a CB-CB distance ≤8Å (CA in the case of Glycine). Ten features were generated for each edge from a total of five types: the distance between the two residues, the angle between the two residues, torsional angles (ω, θ, and ϕ) between the two residues, contact-dependent potential (CDP), and relative solvent accessibility potential (RSAP). CDP and RSAP are only included if the residue pairs are part of the interface.

### 3.2. Training Procedure

Energy-based models (EBMs) are a specific class of deep learning architecture inspired by statistical physics that predict a compatibility score (energy) between a given pair of feature and target vectors. EBMs learn a distribution of choices over an input, as opposed to a single output prediction. Consider the following definitions: G=ExampleSpace, X=FeatureSpace, and Y=TargetSpace. Given an example pair (x,y)∈G, with x∈X & y∈Y, the objective is to maximize EBM(x,t) when t∈Y−{y} and minimize EBM(x,t) when t=y. The ideal EBM would hold the following property ∀(x,y)∈G:

(3) EBM(x,t)=min{EBM(x,a)|∀a∈Y}⇔t=y

During inference, given a fixed feature vector x∈X, the choice of t∈Y that minimizes the energy EBM(x,t) is the predicted target for feature vector *x*.

Training EBMs is computationally expensive, especially for high-dimensional datasets, due to the need to sample and process multiple adversarial inputs per training example. For this reason, we utilized global graph embeddings from pre-trained graph neural networks (GNNs) as input features. The supervised GNNs that produced these embeddings, denoted as pre-trained, were trained separately from the EBMs using a weighted version of the L1 loss function. For details about training the GNNs, see the Supplementary Document (S1).

Our energy-based models (EBMs) were trained using the loss function defined by Equation (4) for energy-based regression [30]. The EBMs were fed fixed global graph embeddings, associated ground truth values, and statistically sampled adversarial target values (logits). Both accuracy scores reside within the space (0,1) and the target values described in [30] reside in the space (−∞,∞). We used the logit function to send accuracy scores to the target space (−∞,∞) and the sigmoid function to return logits to the (0,1) space.

(4) $$J\left(\theta \right)=-\frac{1}{n}\sum_{i=1}^{n}\log\frac{\exp\{f_{\theta }\left(x_{i},y^{\left(i,0\right)}\right)-\log\left(P_{N}\left(y^{\left(i,0\right)}|y_{i}\right)\right\}}{\sum_{m=0}^{M}\exp\{f_{\theta }\left(x_{i},y^{\left(i,m\right)}\right)-\log\left(P_{N}\left(y^{\left(i,m\right)}|y_{i}\right)\right\}}$$

(5) $$P_{N}\left(y|y_{i}\right)=\frac{1}{K}\sum_{K=1}^{K}N\left(y;y_{i},{\sigma_{k}}^{2}I\right)$$

(6) $${\left\{y^{\left(i,m\right)}\right\}}_{m=1}^{M}∼P_{N}\left(y|y_{i}\right)$$

(7) $$P_{\beta }\left(y\right)=\frac{1}{K}\sum_{K=1}^{K}N\left(y;0,\beta {\sigma_{k}}^{2}I\right)$$

(8) $$y^{\left(i,0\right)}≜y_{i}+v_{i},\;v_{i}∼P_{\beta }\left(y\right)$$

In Equation (4), $f_{\theta }$ represents the energy produced by our EBM given an input vector $x_{i}$ and logit *y*. We treated M−1, the number of adversarial (noisy) samples, as a hyper-parameter, and held K=1 as fixed. Equation (8) defines $y^{\left(i,0\right)}$, the true target value $y_{i}$ with additional noise sampled from Equation (7) of intensity β, which is a tunable hyper-parameter. The set of adversarial logits is defined by Equation (6). Adversarial logits are sampled from the normal distribution (5) over the (−∞,∞) space, with the modification of adding a sliding standard deviation (σ) to account for asymptotes in the logit function. The third and first quartiles are positioned at approximately 0.675 standard deviations from the mean in a typical normal distribution. We redefine σ as Equation (9), and define the tunable hyper-parameter γ∈(0,1) that determines σ, given a true target value $y^{i}=\mu$:

(9) $$\sigma =\left\{\begin{array}{cc} \frac{Logit(\mu +\gamma )-Logit(\mu )}{0.675} & \mathrm{if}\;\mu +\gamma <1\\ \frac{Logit(\mu )-Logit(\mu -\gamma )}{0.675} & \mathrm{else} \end{array}\right.$$

The hyper-parameter γ indicates the distance between the quartiles and the mean in the (0,1) space, and (9) determines the approximate σ to achieve this in the (−∞,∞) space. In this way, we hold the normal distributions defined by Equations (5) and (7), which reside in the (−∞,∞) space, generally consistent when sent back to the (0,1) space (using the sigmoid function). Inference through our energy-based models was performed with brute force. Given a fixed global feature vector *X*, we tested logits associated with evenly distributed values in the range (0,1), and chose the logit *Y* that maximized the negative energy (output of our EBMs) as the estimated accuracy of the input protein structure ∼X.

### 3.3. Model Architectures

All model architectures and their respective hyper-parameters were tuned through SHERPA [31] using a genetic algorithm with a mutation rate of 0.1. Refer to the Supplementary Document (S1) for descriptions of the hyper-parameter search space and objective functions utilized by the genetic algorithm. Our models were trained using adaptive gradient clipping with AutoClip [32].

**Figure 6:**
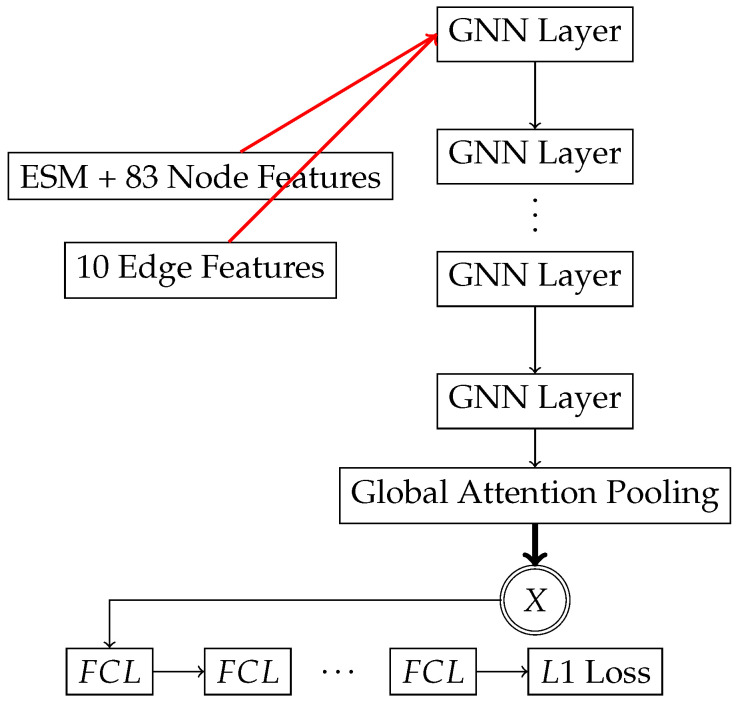
Regression–Transformer and Regression–MetaLayer architectures (pre-trained).

Our pre-trained models utilized GNN layers; see Figure 6. GNN layers are either a graph transformer layer [5] or a MetaLayer [33] followed by ReLU activation. Graph transformer layers were followed by TopKPooling [34,35,36]. Global attention pooling [37] was used to produce the global feature vector *X*. MetaLayer and global attention pooling blocks were composed of fully connected neural networks. A fully connected neural network is used to reduce the global feature vector *X* to the final output prediction. Fully connected layers and GNNs were conditionally (determined by the genetic algorithm) followed by normalization layers [38,39,40].

**Figure 7:**
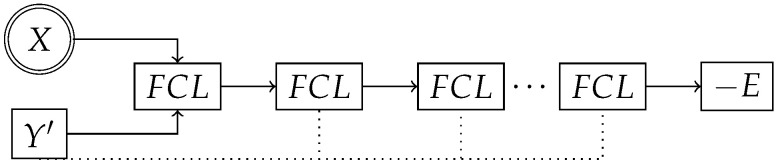
EBM–Transformer and EBM–MetaLayer architectures, which are composed of fully connected layers (FCLs).

Our EBMs were trained separately, and took both the fixed global graph embedding *X* (from the top-performing pre-trained model) and a logit value $Y^{\prime }$ as input. Both inputs *X* and $Y^{\prime }$ are concatenated first before being passed into the first fully connected layer (FCL). Following each FCL are dropout, ReLU activation, and concatenating skip connections (represented as dotted lines) from the input logit $Y^{\prime }$; see Figure 7. We treated the depth of the skip connections as a hyper-parameter. Fully connected layers were conditionally (determined by the genetic algorithm) followed by LayerNorm [39].

The top pre-trained models (GNN Backbones) and EBMs were chosen as the models with the lowest value from the objective function used by the genetic algorithm. Refer to the Supplementary Document (S1) for the descriptions and chosen parameters of the architectures associated with our top-performing models. APOLLO, which was the name of our server participating in CASP15, had a similar architecture to that of our current implementations, but was trained on a smaller dataset.

### 3.4. Implementation

All of our methods were implemented with PyTorch [41] and PyTorch Geometric [42] using NVIDIA A100 GPUs.

## 4. Conclusions

In this work, we developed multiple graph and energy-based architectures for estimating the accuracy of the predicted multimeric protein structures. This task is computationally demanding, and has crucial implications within the medical and biological research fields. Understanding the energy landscape of a protein is analogous to understanding the ways in which the protein can conform in three dimensional space. In theory, the native conformation of a protein would correspond to its lowest free-energy state. Similarly, quality scores inferred from the protein models are attempting to discern a measure of deviance from its corresponding native structure. This work integrates both of these concepts by learning a viable energy function, using deep neural networks, which can directly infer the qualities of individual multimeric protein models. Furthermore, we capitalized on the power of cutting-edge graph neural network architectures to process spatial and topological information advantageously from input protein structures. Our results against the CASP15 targets support the ability of our methods to generalize and produce high-quality insights into the qualities of predicted multimeric protein models.

## Figures and Tables

**Table 1:** Average loss comparison on CASP15 targets.

Type	Backbone	TM-L1	TM-MSE	QS-L1	QS-MSE
Regression	Transformer	0.243	0.094	0.314	0.127
EBM	Transformer	0.245	0.096	0.341	0.156
Regression	MetaLayer	0.263	0.098	0.300	0.124
EBM	MetaLayer	0.242	0.088	0.299	0.126

## Data Availability

EGG is freely available at http://dna.cs.miami.edu/EGG/ (accessed on 1 March 2024) and https://github.com/zwang-bioinformatics/EGG/ (accessed on 1 March 2024).

## Supplementary Materials

The following supporting information can be downloaded at: https://www.mdpi.com/article/10.3390/ijms25116250/s1.

## Abbreviations

The following abbreviations are used in this manuscript:

Multimer	Multiple Chain Protein
Monomer	Single Chain Protein
EMA	Model Accuracy Estimation
CASP	The Critical Assessment of Protein Structure Prediction
GNN	Graph Neural Network
FCL	Fully Connected Layer
EBM	Energy-Based Model
CB	Beta Carbon
CA	Alpha Carbon
CDP	Contact Dependent Potential
RSAP	Relative Solvent Accessibility Potential
MSE	Mean Squared Error
L1	Mean Absolute Error
TM-score	Overall Fold Score
QS-score	Overall Interface Score
DCG	Discounted Cumulative Gain
NDCG	Normalized Discounted Cumulative Gain

## References

[1] Dorn M., e Silva M.B., Buriol L.S., Lamb L.C. (2014). Three-dimensional protein structure prediction: Methods and computational strategies. Comput. Biol. Chem..

[2] Johnson M.S., Srinivasan N., Sowdhamini R., Blundell T.L. (1994). Knowledge-Based Protein Modeling. Crit. Rev. Biochem. Mol. Biol..

[3] Nero T.L., Parker M.W., Morton C.J. (2018). Protein structure and computational drug discovery. Biochem. Soc. Trans..

[4] Jubb H.C., Pandurangan A.P., Turner M.A., Ochoa-Montaño B., Blundell T.L., Ascher D.B. (2017). Mutations at protein-protein interfaces: Small changes over big surfaces have large impacts on human health. Prog. Biophys. Mol. Biol..

[5] Shi Y., Huang Z., Feng S., Zhong H., Wang W., Sun Y. (2020). Masked label prediction: Unified message passing model for semi-supervised classification. arXiv.

[6] Dwivedi V.P., Bresson X. (2020). A Generalization of Transformer Networks to Graphs.

[7] Vaswani A., Shazeer N., Parmar N., Uszkoreit J., Jones L., Gomez A.N., Kaiser L., Polosukhin I. (2017). Attention Is All You Need Advances in Neural Information Processing Systems 30. https://papers.nips.cc/paper_files/paper/2017/hash/3f5ee243547dee91fbd053c1c4a845aa-Abstract.html.

[8] Chen X., Morehead A., Liu J., Cheng J. (2023). A gated graph transformer for protein complex structure quality assessment and its performance in CASP15. Bioinformatics.

[9] Zhao C., Liu T., Wang Z. (2022). Predicting residue-specific qualities of individual protein models using residual neural networks and graph neural networks. Proteins Struct. Funct. Bioinform..

[10] Baldassarre F., Hurtado D.M., Elofsson A., Azizpour H. (2020). GraphQA: Protein model quality assessment using graph convolutional networks. Bioinformatics.

[11] Zhang P., Xia C., Shen H.B. (2022). High-accuracy protein model quality assessment using attention graph neural networks. Briefings Bioinform..

[12] Anfinsen C.B. (1973). Principles that Govern the Folding of Protein Chains. Science.

[13] Alford R.F., Leaver-Fay A., Jeliazkov J.R., O’Meara M.J., DiMaio F.P., Park H., Shapovalov M.V., Renfrew P.D., Mulligan V.K., Kappel K. (2017). The Rosetta All-Atom Energy Function for Macromolecular Modeling and Design. J. Chem. Theory Comput..

[14] Cao Y., Shen Y. (2020). Energy-based graph convolutional networks for scoring protein docking models. Proteins Struct. Funct. Bioinform..

[15] Du Y., Meier J., Ma J., Fergus R., Rives A. (2020). Energy-based models for atomic-resolution protein conformations. arXiv.

[16] Studer G., Tauriello G., Schwede T. (2023). Assessment of the assessment—All about complexes. Proteins Struct. Funct. Bioinform..

[17] Morehead A., Chen X., Wu T., Liu J., Cheng J. (2022). EGR: Equivariant Graph Refinement and Assessment of 3D Protein Complex Structures. arXiv.

[18] Liu J., Liu D., He G., Zhang G. (2023). Estimating protein complex model accuracy based on ultrafast shape recognition and deep learning in CASP15. Proteins Struct. Funct. Bioinform..

[19] Olechnovič K., Venclovas Č. (2023). VoroIF-GNN: Voronoi tessellation-derived protein–protein interface assessment using a graph neural network. Proteins Struct. Funct. Bioinform..

[20] Järvelin K., Kekäläinen J. (2002). Cumulated gain-based evaluation of IR techniques. ACM Trans. Inf. Syst..

[21] (2015). The PyMOL Molecular Graphics System.

[22] Berman H.M. (2000). The Protein Data Bank. Nucleic Acids Res..

[23] Chaudhury S., Lyskov S., Gray J.J. (2010). PyRosetta: A script-based interface for implementing molecular modeling algorithms using Rosetta. Bioinformatics.

[24] Zhang Y., Skolnick J. (2004). Scoring function for automated assessment of protein structure template quality. Proteins Struct. Funct. Bioinform..

[25] Bertoni M., Kiefer F., Biasini M., Bordoli L., Schwede T. (2017). Modeling protein quaternary structure of homo- and hetero-oligomers beyond binary interactions by homology. Sci. Rep..

[26] Rives A., Meier J., Sercu T., Goyal S., Lin Z., Liu J., Guo D., Ott M., Zitnick C.L., Ma J. (2021). Biological structure and function emerge from scaling unsupervised learning to 250 million protein sequences. Proc. Natl. Acad. Sci. USA.

[27] Altschul S. (1997). Gapped BLAST and PSI-BLAST: A new generation of protein database search programs. Nucleic Acids Res..

[28] Liu T., Wang Z. (2018). SOV_refine: A further refined definition of segment overlap score and its significance for protein structure similarity. Source Code Biol. Med..

[29] Liu T., Wang Z. (2020). MASS: Predict the global qualities of individual protein models using random forests and novel statistical potentials. BMC Bioinform..

[30] Gustafsson F.K., Danelljan M., Timofte R., Schön T.B. How to Train Your Energy-Based Model for Regression. Proceedings of the British Machine Vision Conference (BMVC), September, 2022. https://www.bmvc2020-conference.com/assets/papers/0154.pdf.

[31] Hertel L., Collado J., Sadowski P., Ott J., Baldi P. (2020). Sherpa: Robust Hyperparameter Optimization for Machine Learning. SoftwareX.

[32] Seetharaman P., Wichern G., Pardo B., Roux J.L. Autoclip: Adaptive Gradient Clipping for Source Separation Networks. Proceedings of the 2020 IEEE 30th International Workshop on Machine Learning for Signal Processing (MLSP).

[33] Battaglia P., Hamrick J.B.C., Bapst V., Sanchez A., Zambaldi V., Malinowski M., Tacchetti A., Raposo D., Santoro A., Faulkner R. (2018). Relational inductive biases, deep learning, and graph networks. arXiv.

[34] Gao H., Ji S. Graph u-nets. Proceedings of the International Conference on Machine Learning, PMLR.

[35] Cangea C., Veličković P., Jovanović N., Kipf T., Liò P. (2018). Towards sparse hierarchical graph classifiers. arXiv.

[36] Knyazev B., Taylor G.W., Amer M. (2019). Understanding attention and generalization in graph neural networks. Adv. Neural Inf. Process. Syst..

[37] Li Y., Tarlow D., Brockschmidt M., Zemel R. (2015). Gated Graph Sequence Neural Networks. arXiv.

[38] Cai T., Luo S., Xu K., He D., Liu T.Y., Wang L. Graphnorm: A principled approach to accelerating graph neural network training. Proceedings of the International Conference on Machine Learning, PMLR.

[39] Ba J.L., Kiros J.R., Hinton G.E. (2016). Layer Normalization. arXiv.

[40] Ioffe S., Szegedy C. Batch normalization: Accelerating deep network training by reducing internal covariate shift. Proceedings of the International Conference on Machine Learning, PMLR.

[41] Paszke A., Gross S., Massa F., Lerer A., Bradbury J., Chanan G., Killeen T., Lin Z., Gimelshein N., Antiga L. (2019). Pytorch: An imperative style, high-performance deep learning library. Adv. Neural Inf. Process. Syst..

[42] Fey M., Lenssen J.E. Fast Graph Representation Learning with PyTorch Geometric. Proceedings of the ICLR Workshop on Representation Learning on Graphs and Manifolds.

