# Supplementary material for: EGG: Accuracy Estimation of Individual Multimeric Protein Models Using Deep Energy-Based Models and Graph Neural Networks
Source: Int J Mol Sci. 2024 Jun 6;25(11):6250. doi: 10.3390/ijms25116250 (PMC11173161; doi:10.3390/ijms25116250)
Supplement: Supplementary file 1 [file ijms-25-06250-s001.zip › S1.pdf]

Supplementary document (S1) for

EGG: Accuracy Estimation of Individual Multimeric Protein Models Using Deep Energy-Based Models and Graph Neural Networks

Andrew Jordan Siciliano [1,†], Chenguang Zhao [2,†], Tong Liu [1] and Zheng Wang [1,*]

[1] Department of Computer Science, University of Miami, 1365 Memorial Drive, Coral Gables, FL 33124, USA; ajs550@miami.edu (A.J.S.); tong.liu@miami.edu (T.L.)

[2] Computer Information Sciences Department, St. Ambrose University, 518 W. Locust Street, Davenport, IA 52803, USA; zhaochenguang@sau.edu

[*] Correspondence: zheng.wang@miami.edu

[†] These authors contributed equally to this work.

1 Layer Descriptions

1.1 Transformer

The TransformerConv [11] layer, which is implemented by PyTorch Geometric [5], sends node features x to x' using concatenated multi-head attention. Edge feature aggregation for each node sums all neighboring edge features with attention coefficients $\alpha_{i,j}$ as weights. Nodes x_j are in the neighborhood $\mathcal{N}(i)$ of node x_i. Attention coefficients are calculated using key (x_i) and query $(x_j,\, e_{i,j})$ mechanisms. Each attention head has dimension d. β_i was used to learn the optimal mixture of aggregation and skip information.

$$x'_i = \beta_i W_1 x_i + (1 - \beta_i) m_i$$

$$m_i = \sum_{i \in \mathcal{N}(i)} \alpha_{i,j} (W_2 x_j + W_6 e_{i,j})$$

$$\beta_i = \text{sigmoid}(w_5^\top [W_1 x_i, m_i, W_1 x_i - m_i])$$

$$\alpha_{i,j} = \text{softmax}\left(\frac{(W_3 x_i)^\top (W_4 x_j + W_6 e_{i,j})}{\sqrt{d}} \right)$$

1.2 MetaLayer

The MetaLayer [2] is a framework implemented by PyTorch Geometric [5] for defining message passing within an input graph. MetaLayers are composed of node, edge, and global blocks. We only utilized node and edge blocks. Edge blocks update edge features using a neural network $\phi_e(e_{i,j}, x_i, x_j)$ to produce $e'_{i,j}$. Node feature blocks are composed of two steps. First nodes perform a learned mean aggregation of their neighboring nodes and respective updated edge feature information using the neural network $\phi_{n.0}(\{(x_j, e'_{i,j}) | \forall e_{i,j}\})$, followed by PyTorch Geometric's scatter mean over the i'th node's respective edges. This produces the learned aggregated neighborhood of x_i, denoted as $\bar{e}'_i$. The neural network $\phi_{n.1}(x_i, \bar{e}'_i)$ produces the updated node feature x'_i. Our "Node Merge Fully Connected Neural Network" is defined the same as $\phi_{n.0}$ excluding both a preceding edge block and proceeding $\phi_{n.1}$.

1.3 Global Attention Pooling

Global Attention Pooling [10] is implemented by PyTorch Geometric [5] to aggregate node features x into a global graph embedding X using learnable attention scores. N is denoted as the number of nodes in the graph. Attention scores are calculated using the neural network h_{gate} and node features are updated before aggregation using the neural network $h_{\mathbf{\Theta}}$.

$$X = \sum_{n=1}^{N} \text{softmax}\left(h_{\text{gate}}(\mathbf{x}_n)\right) \odot h_{\mathbf{\Theta}}(\mathbf{x}_n)$$

2 Regression Loss Function

Below is the regression loss function $\text{Loss}(n, G)$, with G being a set of example pairs $(x, y) \in G$. We placed each training example in a respective batch into one of n evenly spaced intervals over example space G. $C(y, n)$ defines the category for a $(x, y) \in G$ pair with $y \in [0, 1]$ being the ground truth value and x being the input graph. The number of groups denoted as #Loss Intervals n is a tunable hyper-parameter. For the case where $y = 1$, we manually set y's group to $n - 1$ for group consistency. The loss is computed for each batch $= G$.

$$C(y, n) = \lfloor y * n \rfloor$$
$$\lambda(c, n, G) = \{(x, y) \in G \mid C(y, n) == c\}$$
$$\text{L1}(c, n, G) = \frac{1}{|\lambda(c, n, G)|} \sum_{(x,y) \in \lambda(c,n,G)} \big|P(x) - y\big|$$
$$\text{Loss}(n, G) = \frac{\sum_{c=0}^{n-1} \text{L1}(c, n, G)}{n}$$

3 Genetic Algorithm

The genetic algorithm we used was implemented through SHERPA [7]. The mutation rate was set as 0.1. We trained batches of models in parallel and each model in a successive batch was chosen as a random combination and mutation of the top 33% of currently trained models. The first 10 model architectures were chosen randomly. For each epoch, we ran the objective function and updated the SHERPA [7] study managing the genetic algorithm.

3.1 Objective Function

The objective function is a varient of the regression loss function which utilized average losses from both L1 and MSE loss criteria. First, we placed each example into one of four evenly spaced intervals over example space G using $C(y)$ which defines the category for a $(x, y) \in G$ pair with $y \in [0, 1]$ being the ground truth value and x being the input graph (GNN) or global graph embedding (EBM). In the case where $y = 1$, we manually set y's group to three. We took the average $\text{L1}(x, y)$ and $\text{MSE}(x, y)$ loss of the predicted $P(x)$ and ground truth y for all example pairs $(x, y) \in G$ in category $c \in [0, 3]$ defined by $\lambda(c, G)$. MSE was only used for the energy-based objective function. We then took the average of the losses over all categories to compute the objective function $F(G)$. The objective function is computed once for each epoch with G being the full validation dataset.

$$C(y) = \lfloor y * 4 \rfloor$$
$$\lambda(c, G) = \{(x, y) \in G \mid C(y) == c\}$$
$$\text{L1}(c, G) = \frac{1}{|\lambda(c, G)|} \sum_{(x,y) \in \lambda(c,G)} \big|P(x) - y\big|$$
$$\text{MSE}(c, G) = \frac{1}{|\lambda(c, G)|} \sum_{(x,y) \in \lambda(c,G)} (P(x) - y)^2$$
$$F(G) = \begin{cases} \frac{\sum_{c=0}^{3} \text{MSE}(c,G) + \text{L1}(c,G)}{8} & \text{if Architecture is EBM} \\ \frac{\sum_{c=0}^{3} \text{L1}(c,G)}{4} & \text{if Architecture is GNN} \end{cases}$$

3.2 Tunable Hyper Parameters

All layers are followed by ReLU activation except the final output. Transformer layers concatenated the multi-head attention outputs and set β equal to true. For the neural network parameters, start channels are the first out channels of the initial linear layer, end channels are the out channels of the final linear layer, and channel increment is the difference in channels between each successive layer (excluding the final linear layer's channels). An arbitrary cap is set, based on computational resources, for the maximum layer channel if the increment is positive. The end channels parameter is favored as the final out channel if the number of layers is 1. Skip connections indicate a concatenation of the input of the previous layer to the input of the current layer. If not chosen to optimize through SHERPA [7] the default value for all boolean choices is *False*. For normalization layers and concatenating skip connections ("Classic" means include, "None" means not include): the default would be to not include them. Parameter ranges and choices were chosen experimentally based on computational resources. We conditionally utilized BatchNorm [8], LayerNorm [1], and GraphNorm [3]. Graph transformer layers were followed by TopKPooling [6, 4, 9] and the pooling ratio is a hyperparameter ("None" indicates no Top-K pooling).

3.2.1 Regression-Transformer

Name	Choices/Range
MultiplicativeLR Lambda	[0.1, 1]
Include ESM Stats	{True, False}
Gradient Accumulation	[1, 17]
Autoclip — History Length	[1000, 10000], Inc=500
Learning Rate	[0.0001, 0.001]
Optimizer	{'adamw—0.01', 'adamw—0.1', 'adamw—0.001', 'SGD—0.9', 'SGD—0.99'}
Autoclip — Quartile	[0.05, 0.9]
Training #Loss Intervals	[1, 10]
**Transformer 1**	
Dropout Rate	[0.001, 0.1]
Graph Norm	{'None', 'before', 'after'}
Heads	[1, 4]
Out Channels	[10, 350]
TopKPooling Ratio	{None, 0.6, 0.65, 0.7, 0.75, 0.8, 0.85, 0.9, 0.95}
**Transformer 2**	
Dropout Rate	[0.001, 0.1]
Graph Norm	{'None', 'before', 'after'}
Heads	[1, 8]
Out Channels	[10, 100]
TopKPooling Ratio	{None, 0.6, 0.65, 0.7, 0.75, 0.8, 0.85, 0.9, 0.95}
**Node Merge Fully Connected Neural Network**	
End Channels	[10, 75]
Channel Increment	[-15, -1]
# Layers	[2, 10]
Layer Norm (with affine)	{'before', 'after', 'None'}
Dropout Rate	[0.001, 0.1]
Concatenate Skip Connections	{'Classic', 'None'}
Start Channels	[10, 100]
**Global Attention Pooling**	
h_{Θ} — Channel Increment	[-15, -1]
h_{Θ} — # Layers	[2, 10]
h_{Θ} — Dropout Rate	[0.001, 0.1]
h_{Θ} — Concatenate Skip Connections	{'Classic', 'None'}
h_{Θ} — Start Channels	[10, 50]
h_{Θ} — Layer Norm (with affine)	{'before', 'after', 'None'}
h_{Θ} — End Channels	[10, 50]
h_{gate} — # Layers	[2, 10]
h_{gate} — Start Channels	[10, 50]
h_{gate} — End Channels	1
h_{gate} — Channel Increment	[-15, -1]
h_{gate} — Layer Norm (with affine)	{'before', 'after', 'None'}
h_{gate} — Dropout Rate	[0.001, 0.1]
h_{gate} — Concatenate Skip Connections	{'Classic', 'None'}
**Global Fully Connected Neural Network**	
End Channels	1
Channel Increment	[-15, -1]
# Layers	[2, 10]
Batch Norm (with affine)	{'before', 'after', 'None'}
Dropout Rate	[0.001, 0.1]
Concatenate Skip Connections	{'Classic', 'None'}
Start Channels	[10, 50]

3.2.2 Regression-MetaLayer

Name	Choices/Range
MultiplicativeLR Lambda	[0.1, 1]
Include ESM Stats	{False}
Gradient Accumulation	[1, 17]
Autoclip — History Length	[1000, 10000], Inc=500
Learning Rate	[0.0001, 0.001]
Optimizer	{'adamw—0.01', 'adamw—0.001'}
Autoclip — Quartile	[0.05, 0.9]
Training #Loss Intervals	[1, 10]
MetaLayer	
ϕ_e — # Layers	[2, 4]
ϕ_e — Start Channels	[10, 50]
ϕ_e — End Channels	[10, 25]
ϕ_e — Channel Increment	[-15, -1]
ϕ_e — Dropout Rate	[0.001, 0.01]
$\phi_{n.0}$ — End Channels	[10, 25]
$\phi_{n.0}$ — Start Channels	[10, 50]
$\phi_{n.0}$ — Dropout Rate	[0.001, 0.01]
$\phi_{n.0}$ — # Layers	[2, 4]
$\phi_{n.0}$ — Channel Increment	[-15, -1]
$\phi_{n.1}$ — # Layers	[2, 4]
$\phi_{n.1}$ — Channel Increment	[-15, -1]
$\phi_{n.1}$ — Start Channels	[10, 50]
$\phi_{n.1}$ — Dropout Rate	[0.001, 0.01]
$\phi_{n.1}$ — End Channels	[10, 25]
Node Merge Fully Connected Neural Network	
End Channels	[5, 10]
Channel Increment	[-3, -1]
# Layers	[2, 4]
Layer Norm (with affine)	None
Dropout Rate	[0.001, 0.01]
Concatenate Skip Connections	None
Start Channels	[5, 10]
Global Attention Pooling	
h_Θ — Channel Increment	[-3, -1]
h_Θ — # Layers	[2, 4]
h_Θ — Dropout Rate	[0.001, 0.01]
h_Θ — Concatenate Skip Connections	None
h_Θ — Start Channels	[5, 15]
h_Θ — Layer Norm (with affine)	None
h_Θ — End Channels	[5, 15]
h_{gate} — # Layers	[2, 4]
h_{gate} — Start Channels	[5, 10]
h_{gate} — End Channels	1
h_{gate} — Channel Increment	[-3, -1]
h_{gate} — Layer Norm (with affine)	None
h_{gate} — Dropout Rate	[0.001, 0.01]
h_{gate} — Concatenate Skip Connections	None
Global Fully Connected Neural Network	
End Channels	1
Channel Increment	[-3, -1]
# Layers	[2, 4]
Batch Norm (with affine)	{'before', 'after', 'None'}
Dropout Rate	[0.001, 0.01]
Concatenate Skip Connections	None
Start Channels	[5, 10]

3.2.3 EBM-Transformer

Name	Choices/Range
MultiplicativeLR Lambda	[0.1, 1]
Adversarial Examples	[10, 250]
Depth of Logit Skip Connections	[0.0, 1.0]
β	[0.01, 0.15]
Gradient Accumulation	[1, 4]
Autoclip — History Length	[1000, 10000], Inc=500
Learning Rate	[1e-05, 0.0001]
Optimizer	{'adamw—0.01', 'adamw—0.1', 'adamw—0.001', 'SGD—0.9', 'SGD—0.99'}
Autoclip — Quartile	[0.05, 0.9]
γ	[0.01, 0.1]
Fully Connected Neural Network	
Dropout Rate	[0.01, 0.05]
Channel Increment	[-25, 25]
# Layers	[1, 50]
Layer Norm (with affine)	{'before', 'after', 'None'}
Concatenate Skip Connections	{True, False}
Output Sigmoid	{True, False}
Start Channels	[5, 250]

3.2.4 EBM-MetaLayer

Name	Choices/Range
MultiplicativeLR Lambda	[0.1, 1]
Adversarial Examples	[10, 250]
Depth of Logit Skip Connections	[0.0, 1.0]
β	[0.01, 0.15]
Gradient Accumulation	[1, 4]
Autoclip — History Length	[1000, 10000], Inc=500
Learning Rate	[1e-05, 0.0001]
Optimizer	{'adamw—0.01', 'adamw—0.1', 'adamw—0.001', 'SGD—0.9', 'SGD—0.99'}
Autoclip — Quartile	[0.05, 0.9]
γ	[0.01, 0.1]
Fully Connected Neural Network	
Dropout Rate	[0.01, 0.05]
Channel Increment	[-5, 5]
# Layers	[1, 25]
Layer Norm (with affine)	{'before', 'after', 'None'}
Concatenate Skip Connections	{True, False}
Output Sigmoid	{True, False}
Start Channels	[4, 35]

4 Model Architectures

4.1 TM-Regression-Transformer

Name	Choices/Range
MultiplicativeLR Lambda	0.9906244444217895
Include ESM Stats	False
Gradient Accumulation	1
Autoclip — History Length	8000
Learning Rate	0.0007474212917477586
Optimizer	adamw—0.1
Autoclip — Quartile	0.3304481962726615
Training #Loss Intervals	8
Transformer 1	
Dropout Rate	0.0702853237265109
Graph Norm	None
Heads	2
Out Channels	184
TopKPooling Ratio	None
Transformer 2	
Dropout Rate	0.020714506064862366
Graph Norm	before
Heads	5
Out Channels	16
TopKPooling Ratio	0.75
Node Merge Fully Connected Neural Network	
End Channels	27
Channel Increment	-14
# Layers	2
Layer Norm (with affine)	before
Dropout Rate	0.06865718729943136
Concatenate Skip Connections	Classic
Start Channels	42
Global Attention Pooling	
h_Θ — Channel Increment	-14
h_Θ — # Layers	4
h_Θ — Dropout Rate	0.025014060284290162
h_Θ — Concatenate Skip Connections	None
h_Θ — Start Channels	37
h_Θ — Layer Norm (with affine)	None
h_Θ — End Channels	43
h_{gate} — # Layers	8
h_{gate} — Start Channels	28
h_{gate} — End Channels	1
h_{gate} — Channel Increment	-10
h_{gate} — Layer Norm (with affine)	None
h_{gate} — Dropout Rate	0.03590396455852002
h_{gate} — Concatenate Skip Connections	None
Global Fully Connected Neural Network	
End Channels	1
Channel Increment	-15
# Layers	6
Batch Norm (with affine)	None
Dropout Rate	0.08787026936883877
Concatenate Skip Connections	Classic
Start Channels	14

4.2 QS-Regression-Transformer

Name	Choices/Range
MultiplicativeLR Lambda	0.716070336557133
Include ESM Stats	False
Gradient Accumulation	6
Autoclip — History Length	7000
Learning Rate	0.0002673542192474871
Optimizer	adamw—0.001
Autoclip — Quartile	0.20758013386612512
Training #Loss Intervals	2
Transformer 1	
Dropout Rate	0.04421118086861678
Graph Norm	before
Heads	3
Out Channels	335
TopKPooling Ratio	0.75
Transformer 2	
Dropout Rate	0.04944193080500327
Graph Norm	before
Heads	4
Out Channels	22
TopKPooling Ratio	0.95
Node Merge Fully Connected Neural Network	
End Channels	71
Channel Increment	-6
# Layers	7
Layer Norm (with affine)	before
Dropout Rate	0.0638053022452135
Concatenate Skip Connections	None
Start Channels	98
Global Attention Pooling	
h_Θ — Channel Increment	-11
h_Θ — # Layers	5
h_Θ — Dropout Rate	0.09013665213482475
h_Θ — Concatenate Skip Connections	None
h_Θ — Start Channels	19
h_Θ — Layer Norm (with affine)	before
h_Θ — End Channels	46
h_{gate} — # Layers	6
h_{gate} — Start Channels	46
h_{gate} — End Channels	1
h_{gate} — Channel Increment	-8
h_{gate} — Layer Norm (with affine)	after
h_{gate} — Dropout Rate	0.029833993342711772
h_{gate} — Concatenate Skip Connections	None
Global Fully Connected Neural Network	
End Channels	1
Channel Increment	-10
# Layers	3
Batch Norm (with affine)	before
Dropout Rate	0.04996441372661872
Concatenate Skip Connections	None
Start Channels	45

4.3 TM-Regression-MetaLayer

Name	Choices/Range
MultiplicativeLR Lambda	0.8837524695301462
Include ESM Stats	False
Gradient Accumulation	15
Autoclip — History Length	8500
Learning Rate	0.00045377140779419837
Optimizer	adamw—0.01
Autoclip — Quartile	0.7638161120157557
Training #Loss Intervals	7
MetaLayer	
ϕ_e — # Layers	3
ϕ_e — Start Channels	16
ϕ_e — End Channels	19
ϕ_e — Channel Increment	-13
ϕ_e — Dropout Rate	0.005230199106083582
$\phi_{n.0}$ — End Channels	23
$\phi_{n.0}$ — Start Channels	13
$\phi_{n.0}$ — Dropout Rate	0.009043754416465776
$\phi_{n.0}$ — # Layers	2
$\phi_{n.0}$ — Channel Increment	-8
$\phi_{n.1}$ — # Layers	3
$\phi_{n.1}$ — Channel Increment	-10
$\phi_{n.1}$ — Start Channels	10
$\phi_{n.1}$ — Dropout Rate	0.007674440275016707
$\phi_{n.1}$ — End Channels	14
Node Merge Fully Connected Neural Network	
End Channels	5
Channel Increment	-2
# Layers	2
Layer Norm (with affine)	None
Dropout Rate	0.00951847744246851
Concatenate Skip Connections	None
Start Channels	5
Global Attention Pooling	
h_Θ — Channel Increment	-3
h_Θ — # Layers	3
h_Θ — Dropout Rate	0.0043462144563076646
h_Θ — Concatenate Skip Connections	None
h_Θ — Start Channels	13
h_Θ — Layer Norm (with affine)	None
h_Θ — End Channels	13
h_{gate} — # Layers	3
h_{gate} — Start Channels	6
h_{gate} — End Channels	1
h_{gate} — Channel Increment	-3
h_{gate} — Layer Norm (with affine)	None
h_{gate} — Dropout Rate	0.001962812482182936
h_{gate} — Concatenate Skip Connections	None
Global Fully Connected Neural Network	
End Channels	1
Channel Increment	-3
# Layers	2
Batch Norm (with affine)	None
Dropout Rate	0.006566923513910563
Concatenate Skip Connections	None
Start Channels	9

4.4 QS-Regression-MetaLayer

Name	Choices/Range
MultiplicativeLR Lambda	0.9527601973485689
Include ESM Stats	False
Gradient Accumulation	5
Autoclip — History Length	1500
Learning Rate	0.00028365658616461936
Optimizer	adamw—0.001
Autoclip — Quartile	0.6440493330813005
Training #Loss Intervals	2
MetaLayer	
ϕ_e — # Layers	3
ϕ_e — Start Channels	10
ϕ_e — End Channels	12
ϕ_e — Channel Increment	-8
ϕ_e — Dropout Rate	0.007249390294281908
$\phi_{n.0}$ — End Channels	24
$\phi_{n.0}$ — Start Channels	37
$\phi_{n.0}$ — Dropout Rate	0.006553663984072305
$\phi_{n.0}$ — # Layers	3
$\phi_{n.0}$ — Channel Increment	-10
$\phi_{n.1}$ — # Layers	2
$\phi_{n.1}$ — Channel Increment	-4
$\phi_{n.1}$ — Start Channels	35
$\phi_{n.1}$ — Dropout Rate	0.008012210164819171
$\phi_{n.1}$ — End Channels	18
Node Merge Fully Connected Neural Network	
End Channels	6
Channel Increment	-3
# Layers	2
Layer Norm (with affine)	None
Dropout Rate	0.006187360485172214
Concatenate Skip Connections	None
Start Channels	9
Global Attention Pooling	
h_Θ — Channel Increment	-2
h_Θ — # Layers	3
h_Θ — Dropout Rate	0.0018288389000533827
h_Θ — Concatenate Skip Connections	None
h_Θ — Start Channels	14
h_Θ — Layer Norm (with affine)	None
h_Θ — End Channels	12
h_{gate} — # Layers	2
h_{gate} — Start Channels	7
h_{gate} — End Channels	1
h_{gate} — Channel Increment	-2
h_{gate} — Layer Norm (with affine)	None
h_{gate} — Dropout Rate	0.0073500484211210665
h_{gate} — Concatenate Skip Connections	None
Global Fully Connected Neural Network	
End Channels	1
Channel Increment	-2
# Layers	3
Batch Norm (with affine)	after
Dropout Rate	0.0033415329355790512
Concatenate Skip Connections	None
Start Channels	5

4.5 TM-EBM-Transformer

Name	Choices/Range
MultiplicativeLR Lambda	0.6308857940275763
Adversarial Examples	225
Depth of Logit Skip Connections	0.17862162362431055
β	0.07206053807700703
Gradient Accumulation	2
Autoclip — History Length	7000
Learning Rate	3.677917782778102e-05
Optimizer	adamw—0.001
Autoclip — Quartile	0.8458320295226648
γ	0.07378176895758758
Fully Connected Neural Network	
Dropout Rate	0.013638975193753877
Channel Increment	19
# Layers	6
Layer Norm (with affine)	None
Concatenate Skip Connections	False
Output Sigmoid	True
Start Channels	38

4.6 QS-EBM-Transformer

Name	Choices/Range
MultiplicativeLR Lambda	0.47921293243966123
Adversarial Examples	95
Depth of Logit Skip Connections	0.19199646931029346
β	0.12037915731617012
Gradient Accumulation	1
Autoclip — History Length	7000
Learning Rate	4.942581886452797e-05
Optimizer	adamw—0.01
Autoclip — Quartile	0.5006764299225214
γ	0.08515378879892439
Fully Connected Neural Network	
Dropout Rate	0.03640975336429724
Channel Increment	15
# Layers	18
Layer Norm (with affine)	before
Concatenate Skip Connections	True
Output Sigmoid	True
Start Channels	105

4.7 TM-EBM-MetaLayer

Name	Choices/Range
MultiplicativeLR Lambda	0.675302826696286
Adversarial Examples	143
Depth of Logit Skip Connections	0.29402334023232024
β	0.05094308763039647
Gradient Accumulation	2
Autoclip — History Length	2500
Learning Rate	1.9689939116937563e-05
Optimizer	adamw—0.1
Autoclip — Quartile	0.538130052912375
γ	0.05079317405215908
Fully Connected Neural Network	
Dropout Rate	0.011843496051657914
Channel Increment	-5
# Layers	15
Layer Norm (with affine)	after
Concatenate Skip Connections	False
Output Sigmoid	False
Start Channels	28

4.8 QS-EBM-MetaLayer

Name	Choices/Range
MultiplicativeLR Lambda	0.13401759804605012
Adversarial Examples	106
Depth of Logit Skip Connections	0.25473669581538916
β	0.06896271729898369
Gradient Accumulation	2
Autoclip — History Length	1500
Learning Rate	6.424327445445079e-05
Optimizer	adamw—0.1
Autoclip — Quartile	0.3509564287988935
γ	0.09128038957755642
Fully Connected Neural Network	
Dropout Rate	0.02265478782878598
Channel Increment	-5
# Layers	17
Layer Norm (with affine)	before
Concatenate Skip Connections	True
Output Sigmoid	True
Start Channels	6

References

[1] Jimmy Lei Ba, Jamie Ryan Kiros, and Geoffrey E. Hinton. Layer normalization, 2016.

[2] Peter Battaglia, Jessica Blake Chandler Hamrick, Victor Bapst, Alvaro Sanchez, Vinicius Zambaldi, Mateusz Malinowski, Andrea Tacchetti, David Raposo, Adam Santoro, Ryan Faulkner, Caglar Gulcehre, Francis Song, Andy Ballard, Justin Gilmer, George E. Dahl, Ashish Vaswani, Kelsey Allen, Charles Nash, Victoria Jayne Langston, Chris Dyer, Nicolas Heess, Daan Wierstra, Pushmeet Kohli, Matt Botvinick, Oriol Vinyals, Yujia Li, and Razvan Pascanu. Relational inductive biases, deep learning, and graph networks. *arXiv*, 2018.

[3] Tianle Cai, Shengjie Luo, Keyulu Xu, Di He, Tie-yan Liu, and Liwei Wang. Graphnorm: A principled approach to accelerating graph neural network training. In *International Conference on Machine Learning*, pages 1204–1215. PMLR, 2021.

[4] Cătălina Cangea, Petar Veličković, Nikola Jovanović, Thomas Kipf, and Pietro Liò. Towards sparse hierarchical graph classifiers. *arXiv preprint arXiv:1811.01287*, 2018.

[5] Matthias Fey and Jan E. Lenssen. Fast graph representation learning with PyTorch Geometric. In *ICLR Workshop on Representation Learning on Graphs and Manifolds*, 2019.

[6] Hongyang Gao and Shuiwang Ji. Graph u-nets. In *international conference on machine learning*, pages 2083–2092. PMLR, 2019.

[7] Lars Hertel, Julian Collado, Peter Sadowski, Jordan Ott, and Pierre Baldi. Sherpa: Robust hyperparameter optimization for machine learning. *SoftwareX*, 2020. In press.

[8] Sergey Ioffe and Christian Szegedy. Batch normalization: Accelerating deep network training by reducing internal covariate shift. In *International conference on machine learning*, pages 448–456. pmlr, 2015.

[9] Boris Knyazev, Graham W Taylor, and Mohamed Amer. Understanding attention and generalization in graph neural networks. *Advances in neural information processing systems*, 32, 2019.

[10] Yujia Li, Daniel Tarlow, Marc Brockschmidt, and Richard Zemel. Gated graph sequence neural networks, 2015.

[11] Yunsheng Shi, Zhengjie Huang, Shikun Feng, Hui Zhong, Wenjin Wang, and Yu Sun. Masked label prediction: Unified message passing model for semi-supervised classification. *arXiv preprint arXiv:2009.03509*, 2020.
